# Nationwide analysis of temporal trends and outcomes in hospitalized patients with predominantly antibody deficiency using the National Inpatient Sample

**DOI:** 10.1016/j.jacig.2026.100691

**Published:** 2026-03-20

**Authors:** Ahmed Elmoursi, Lingxiao Zhang, Sara Barmettler

**Affiliations:** a Division of Rheumatology, Allergy, and Immunology, Department of Medicine, Massachusetts General Hospital, Boston, Mass; b Harvard Medical School, Boston, Mass

**Keywords:** Predominantly antibody deficiency, primary immunodeficiency, National Inpatient Sample, hospitalization outcomes, temporal trends, health care utilization, inborn errors of immunity, mortality, hospital cost, hospital stay

## Abstract

**Background:**

Predominantly antibody deficiency (PAD) is the most common inborn error of immunity and is associated with increased susceptibility to infections and noninfectious complications, yet we lack population-level data on the impact of this diagnosis on hospitalization outcomes.

**Objective:**

We conducted a nationwide analysis evaluating health care utilization and hospitalization outcomes among patients with PAD compared with non-PAD hospitalized patients, examining temporal trends and the impact of coronavirus disease 2019.

**Methods:**

We performed a cross-sectional analysis from 2017 to 2020 using the National Inpatient Sample, part of the Healthcare Cost and Utilization Project by the Agency for Healthcare Research and Quality. ICD-10 codes were used to identify PAD-associated admissions.

**Results:**

We identified 179,710 weighted PAD-associated and 108,933,035 non-PAD–associated hospitalizations. Compared with non-PAD admissions, PAD hospitalizations had higher total hospital costs ($13,961 vs $9,436), longer length of stay (5.0 vs 3.5 days), and higher in-hospital mortality (4.1% vs 2.7%; all *P* < .0001). In adjusted survey-weighted models, PAD remained independently associated with substantially higher costs and longer hospital stays, without increased odds of in-hospital mortality. Temporal analysis from 2017 to 2020 revealed increasing costs ($12,611 to $15,373), length of stay (4.4 to 4.6 days), and in-hospital mortality (3.8% to 4.5%; all *P* < .0001).

**Conclusions:**

Patients with PAD had increased health care utilization and worse outcomes including in-hospital mortality. These burdens worsened over time, with the greatest escalation in 2020, partly reflecting coronavirus disease 2019–related hospitalizations. Further investigation is needed to identify underlying drivers and develop targeted strategies to improve outcomes in patients with PAD.

Predominantly antibody deficiency (PAD) is the most frequently diagnosed category of inborn errors of immunity and the most common form of primary immunodeficiency.^1^, ^2^, ^3^, ^4^, ^5^ PAD is characterized by impaired antibody production or function and recurrent infections, particularly involving the respiratory and gastrointestinal tracts.^6^^,^^7^ Beyond infection-related morbidity, PAD is associated with systemic, noninfectious complications including autoimmunity, interstitial lung disease, lymphoproliferative disorders, and malignancy, all of which contribute to a significant long-term health burden and reduced life expectancy.^3^

Common forms of PAD include common variable immunodeficiency (CVID), X-linked agammaglobulinemia (XLA), and selective IgA deficiency (SIGAD).^2^^,^^3^^,^^5^ Clinical presentations vary in age at onset and severity, ranging from asymptomatic cases such as many individuals with IgA deficiency to conditions like CVID and XLA, which are associated with substantial morbidity.^1^, ^2^, ^3^ Patients with symptomatic PAD often require lifelong immunoglobulin replacement therapy to reduce infection frequency and prevent long-term complications in addition to monitoring and treating noninfectious complications.^1^^,^^4^^,^^6^

Despite advances in diagnosis and therapy, individuals with PAD remain at elevated risk for hospitalization due to both infectious and systemic complications. Bronchiectasis, for instance, is present in approximately 34% of patients with CVID and often necessitates inpatient care.^8^ Autoimmune manifestations, including cytopenias and arthritis, affect up to 18% and 11% of CVID patients, respectively, and frequently lead to hospitalization during disease flares.^9^ Gastrointestinal involvement, such as protein-losing enteropathy and malabsorption, affects up to 50% of CVID patients and can result in severe metabolic derangements requiring inpatient treatment.^10^ Malignancies, particularly non-Hodgkin lymphoma, are the most frequently observed cancers in PAD and contribute significantly to the hospitalization burden.^11^^,^^12^ These overlapping comorbidities highlight the multisystemic nature of PAD and its substantial impact on health care utilization.

Hospitalization in PAD often reflects the cumulative effects of delayed diagnosis, chronic immune dysregulation, and complications involving multiple organ systems. Several studies have explored hospitalization patterns through single-center cohorts or disease-specific registries. For example, the BIPAD (Burden of Infection in Primary Antibody Deficiency) study from Wales identified respiratory infections as the most frequent cause of admission,^13^ while analyses from the United States Immunodeficiency Network and the European Society for Immunodeficiencies registries underscored the roles of infections, autoimmunity, and chronic lung disease in driving hospital utilization.^9^^,^^14^^,^^15^ However, these data are limited to selected populations and do not provide a comprehensive national perspective.

Key hospitalization metrics such as total cost, length of stay (LOS), discharge disposition, and in-hospital mortality remain poorly characterized in PAD. Furthermore, how these outcomes compare to the general inpatient population or how they have changed over time, particularly in the setting of the coronavirus disease 2019 (COVID-19) pandemic, is not well understood.

Our study aimed to address these knowledge gaps by conducting a nationally representative evaluation of hospitalization patterns and outcomes among adults with PAD using the National Inpatient Sample (NIS) from 2017 to 2020.^16^ We compared total hospital costs, LOS, discharge disposition, and in-hospital mortality between PAD and non-PAD hospitalizations and examined temporal trends across the study period. To strengthen causal inference and address potential misclassification, we incorporated several sensitivity and stratified analyses, including restriction to subgroups with CVID and XLA and assessment of outcomes across principal clinical diagnosis categories.

Given the emergence of COVID-19 in 2020, we further differentiated PAD hospitalizations with and without COVID-19, examined respiratory principal diagnoses, and tested for interaction between PAD status and COVID-19 to evaluate whether the pandemic modified PAD-associated risk. Leveraging one of the largest all-payer inpatient datasets in the United States, this study provides the most comprehensive national assessment to date of hospitalization burden in PAD and offers insight to inform clinicians, researchers, and policymakers in identifying high-risk subgroups, guiding resource allocation, and developing targeted strategies to improve outcomes in this vulnerable population.

## Methods

We conducted a retrospective cross-sectional analysis using nationally representative hospital discharge data from the NIS from 2017 through 2020. The NIS is part of the Healthcare Cost and Utilization Project, sponsored by the Agency for Healthcare Research and Quality, and represents the largest publicly available, all-payer inpatient database in the United States.^16^ It provides nationally representative estimates of hospitalizations from nonfederal, acute-care hospitals and includes data on approximately 7 million discharges per year, which can be weighted to estimate more than 35 million hospitalizations annually.^16^

The NIS is derived from a stratified probability sample of hospitals that is based on characteristics such as region, location (urban/rural), ownership, teaching status, and bed size. Each discharge record includes information on patient demographics, diagnoses, procedures, discharge disposition, LOS, in-hospital mortality, and total charges. Diagnoses and procedures are coded using the International Classification of Diseases, Tenth Revision, Clinical Modification/Procedure Coding System (ICD-10-CM/PCS). Cost estimates were derived by applying Healthcare Cost and Utilization Project–provided hospital-specific cost-to-charge ratios to total hospital charges. This study used deidentified, publicly available data and was deemed exempt from institutional review board approval.^16^

We identified hospitalizations associated with PAD by using ICD-10-CM diagnosis codes for conditions including CVID (D83.x), XLA (D80.0), SIGAD (D80.2), and other related antibody deficiencies (D80.x). We included all adult hospitalizations (age ≥ 18 years) with any PAD code listed as a principal or secondary diagnosis from 2017 to 2020. Non-PAD–associated admissions were defined as all other adult inpatient discharges without a PAD diagnosis during the same time frame.

Elective admissions and hospitalizations with missing demographic or outcome data were excluded. We also excluded obstetric encounters and applied age and sex standardization. To evaluate the impact of elective admission exclusions, we conducted a sensitivity analysis repeating all comparisons with elective admissions retained. Because PAD codes in nonprincipal positions may reflect historical or incidental diagnoses, we conducted additional analyses restricted to hospitalizations with CVID (D83.x) or XLA (D80.0) as the PAD subtype and further stratified PAD admissions into CVID/XLA, SIGAD, and other PAD groups.

We assessed 4 primary hospitalization outcomes included total hospitalization cost (adjusted to 2020 US dollars using the Consumer Price Index), LOS (in days), discharge disposition (routine, skilled nursing facility, home health care, against medical advice, or in-hospital death), and in-hospital mortality. Principal diagnosis categories were evaluated to characterize clinical reasons for hospitalization among PAD patients and to determine whether outcomes differed by major clinical categories. We compared outcomes between PAD and non-PAD admissions and assessed trends over time from 2017 to 2020.

To evaluate the impact of the COVID-19 pandemic, we examined 2020 hospitalizations with and without a COVID-19 diagnosis (ICD-10 U07.1) and compared outcomes within PAD and non-PAD cohorts. We additionally evaluated admissions with a respiratory principal diagnosis to assess the contribution of respiratory disease to 2020 outcomes. To test whether the relationship between PAD and outcomes (mortality, LOS, and cost) differed by COVID-19 status, we included PAD × COVID-19 interaction terms in adjusted models.

Patient-level covariates included age, sex, and race/ethnicity. While patient-level data are available, the NIS does not track patients year to year, so the data do not allow analysis of readmissions or longitudinal progress of an individual patient. Descriptive statistics were used to summarize patient and hospitalization characteristics. Categorical variables, including sex, race, discharge disposition, and vital status, were compared by survey-weighted chi-square tests implemented in PROC SURVEYFREQ, accounting for the NIS sampling design. Continuous variables were summarized as medians with interquartile ranges (IQRs) and compared by a survey-weighted extension of the Mood median test that incorporates NIS weights, strata, and clustering.

Multivariable, survey-weighted regression models were constructed. In-hospital mortality was analyzed by survey-weighted logistic regression, adjusting for age, sex, race/ethnicity, payer, hospital bed size, hospital location and teaching status, census region, elective admission status, and Elixhauser comorbidity burden. LOS and total hospital costs were analyzed by generalized linear models with a gamma distribution and log link, appropriate for skewed utilization outcomes. A 2-sided *P* value of <.05 was considered statistically significant. All statistical analyses were conducted by SAS v9.4 (SAS Institute, Cary, NC).

## Results

### PAD vs non-PAD hospitalizations

A total of 35,942 unweighted PAD hospitalizations (weighted n = 179,710) were identified from 2017 to 2020 after excluding obstetric encounters and applying age and sex standardization. The comparison group included 21,786,610 unweighted non-PAD hospitalizations (weighted n = 108,933,035) (Table I). Patients with PAD were slightly younger than those without PAD, with an average age of 57.8 ± 0.4 years compared with 59.4 ± 0.1 years in the non-PAD group (*P* < .0001). Sex distribution was similar across groups, with PAD admissions comprising 49.5% male (n = 88,956) and 50.5% female (n = 90,754) subjects—identical to the distribution observed in non-PAD admissions (49.5% male, n = 53,921,852; 50.5% female, n = 55,011,183; *P* = 1.000).

**Table I:** Demographic and hospitalization outcomes among PAD and non-PAD admissions, excluding obstetric admissions and standardized by age and sex (2017-20)

Characteristic	PAD admissions	Non-PAD admissions	*P* value
No., unweighted (weighted)	35,942 (179,710)	21,786,610 (108,933,035)	
Age (years), average [standard error]	57.8 [0.4]	59.4 [0.1]	<.0001
Sex			1.0000
Male	49.5 (88,956)	49.5 (53,921,852)	
Female	50.5 (90,754)	50.5 (55,011,183)	
Race			<.0001
Non-Hispanic White	81.9 (147,182)	66.1 (72,004,736)	
Black/African American	5.1 (9,165)	15.0 (16,339,955)	
Hispanic	6.1 (10,962)	10.3 (11,220,103)	
Other	4.1 (7,368)	5.8 (6,318,116)	
Total hospital cost (US$), median (IQR)	13,961 (7,715-28,438)	9,436 (5,414-16,943)	<.0001
LOS (days), median (IQR)	5.0 (2.8-9.3)	3.5 (2.0-5.8)	<.0001
Discharge disposition			<.0001
Routine	58.1 (104,412)	59.6 (64,924,089)	
Short-term hospital	2.1 (3,774)	2.2 (2,395,627)	
Transfer	14.0 (25,159)	17.5 (19,063,281)	
Home health care	21.2 (38,100)	16.2 (17,641,152)	
Against medical advice	0.6 (1,078)	1.8 (1,960,795)	
Died	4.1 (7,368)	2.7 (2,941,192)	
Vital status			<.0001
Alive	95.9 (172,342)	97.3 (105,991,843)	
Deceased	4.1 (7,368)	2.7 (2,941,192)	

Data are presented as % (nos.) unless otherwise indicated.

Racial and ethnic composition differed significantly between groups (*P* < .0001) (Table I). Among PAD hospitalizations, 81.9% (n = 147,182) were non-Hispanic White, 5.1% (n = 9,615) were Black or African American, 6.1% (n = 10,962) were Hispanic, and 4.1% (n = 7,368) identified as other or unknown race. In contrast, non-PAD hospitalizations were 66.1% (n = 72,004,736) non-Hispanic White, 15.0% (n = 16,339,955) Black or African American, 10.3% (n = 11,220,103) Hispanic, and 5.8% (n = 6,318,116) other or unknown race.

**Fig 1:**
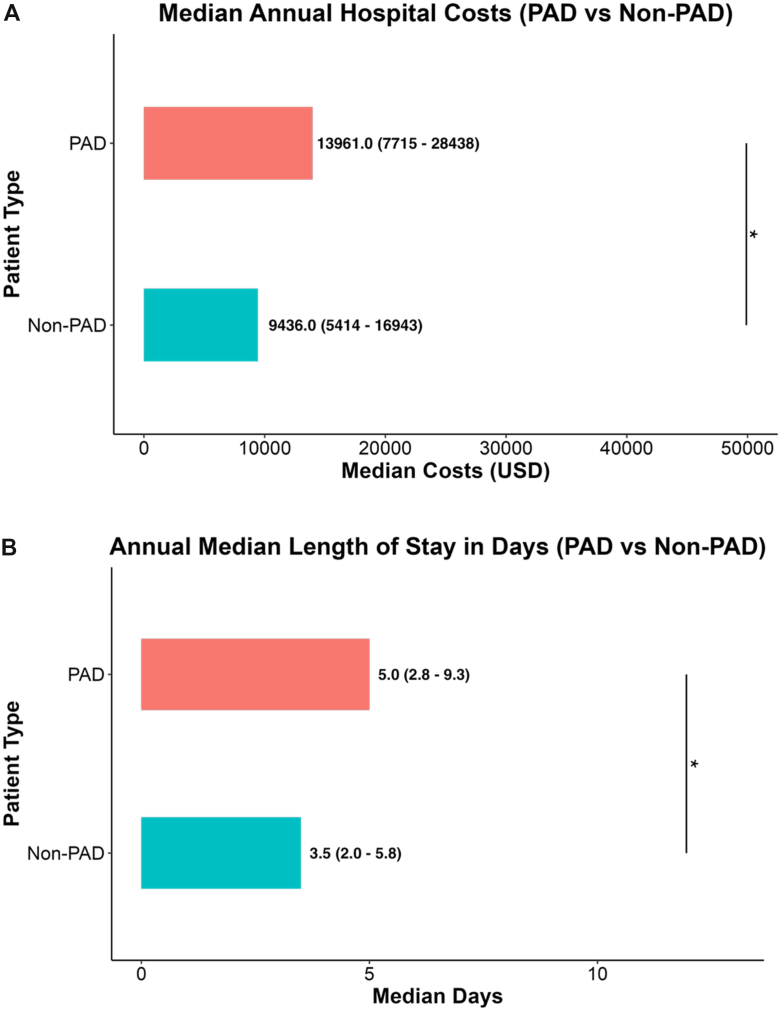
Increased health care utilization among PAD compared with non-PAD hospitalizations. **A,** Median total hospital cost was significantly higher among PAD compared with non-PAD hospitalizations ($13,961 [IQR, $7,715 to $28,438] vs $9,436 [IQR, $5,414 to $16,943]; *P* < .0001). **B,** PAD-associated admissions were associated with significantly longer median [IQR] LOS (5.0 [2.8-9.3] days vs 3.5 [2.0-5.8] days; *P* < .0001). Values are reported as medians with IQRs. ∗*P* < .0001.

Hospitalizations associated with PAD were characterized by significantly greater health care utilization compared with non-PAD admissions (Table I, Fig 1). Median total hospital costs were substantially higher among PAD hospitalizations, with a median cost of $13,961 (IQR, $7,715 to $28,438) compared with $9,436 (IQR, $5,414 to $16,943) in non-PAD admissions (*P* < .0001) (Fig 1, *A*). Similarly, median LOS was significantly longer for PAD patients, who remained hospitalized for a median of 5.0 days (IQR, 2.8-9.3) compared with 3.5 days (IQR, 2.0-5.8) in the non-PAD group (*P* < .0001) (Fig 1, *B*).

Discharge disposition patterns also differed significantly between groups (*P* < .0001) (Table I; see Fig E1 in the Online Repository available at www.jaci-global.org). Routine discharge was less common among PAD hospitalizations, occurring in 58.1% (n = 104,412) of admissions, compared with 59.6% (n = 64,924,089) in non-PAD hospitalizations. PAD patients more frequently required home health care services, with 21.2% (n = 38,100) discharged to home health care compared with 16.2% (n = 17,641,152) in the non-PAD group. Transfers to other facilities were also common among PAD admissions (14.0%, n = 25,159), although slightly less frequent than in non-PAD admissions (17.5%, n = 19,063,281). Discharges against medical advice were rare in the PAD cohort (0.6%, n = 1,078) and occurred less frequently than in non-PAD admissions (1.8%, n = 1,960,795) (Fig E1).

In-hospital mortality was significantly higher among PAD hospitalizations, occurring in 4.1% (n = 7,368) of admissions compared with 2.7% (n = 2,941,192) among non-PAD hospitalizations (*P* < .0001). Correspondingly, PAD patients were less frequently discharged alive (95.9%, n = 172,342) than non-PAD patients (97.3%, n = 105,991,843) (Table I). Infectious diagnoses accounted for 3,090 deaths (5.18% mortality; *P* = .582), autoinflammatory/autoimmune conditions for 45 deaths (2.62% mortality; *P* < .0001), and other principal diagnoses for 3,750 deaths (3.12% mortality; *P* < .0001).

### Adjusted hospitalization outcomes and sensitivity analyses

A sensitivity analysis was performed to evaluate the impact of excluding elective admissions on cohort composition and hospitalization outcomes (see Table E1 in the Online Repository available at www.jaci-global.org). Elective hospitalizations represented a small proportion of overall admissions in both groups, accounting for 142 unweighted admissions (weighted n = 5,177; 2.9%) in PAD and 3,645 unweighted admissions (weighted n = 154,349; 5.5%) in the non-PAD cohort (*P* < .0001). After exclusion, the final analytic sample consisted of 31,119 unweighted PAD admissions (weighted n = 155,595) and 22,269,537 unweighted non-PAD admissions (weighted n = 113,476,666).

When elective admissions were retained (Table E1), PAD hospitalizations continued to demonstrate higher median hospital costs ($13,961; IQR, $7,479 to $27,154) compared with non-PAD admissions ($9,520; IQR, $4,324 to $14,580; *P* < .0001). Median LOS also remained longer among PAD admissions (4.4 days; IQR, 2.2-8.6) relative to non-PAD hospitalizations (2.4 days; IQR, 1.3-4.7; *P* < .0001). Similarly, in-hospital mortality was consistently elevated in PAD hospitalizations whether elective encounters were included (3.8% vs 2.2%; *P* < .0001) or excluded (4.1% vs 2.7%; *P* < .0001).

To address potential confounding from demographic, clinical, and hospital-level differences, we performed multivariable, survey-weighted regression analyses adjusting for age, sex, race/ethnicity, payer, hospital region, hospital bed size, hospital location and teaching status, census region, elective admission status, and Elixhauser comorbidity burden (see Table E2 in the Online Repository available at www.jaci-global.org). PAD remained independently associated with significantly greater health care utilization after multivariable adjustment. PAD hospitalizations demonstrated a longer adjusted LOS, with a GLM (generalized linear model) gamma/log coefficient of 0.537 (95% confidence interval [CI], 0.528-0.546; *P* < .0001). Similarly, total hospital costs were significantly higher among PAD admissions, with a gamma/log coefficient of 0.938 (95% CI, 0.928-0.948; *P* < .0001) (Table E2). In contrast, PAD status was associated with lower odds of in-hospital mortality compared with non-PAD hospitalizations (adjusted odds ratio = 0.738; 95% CI, 0.694-0.785; *P* < .0001) (Table E2).

### Sensitivity analyses by PAD subtype

To address the possibility that PAD codes may reflect historical or incidental diagnoses when listed in secondary positions, we performed additional analyses restricted to patients with CVID or XLA (Table II). This CVID/XLA-restricted cohort included 6,981 unweighted admissions (weighted n = 34,905). Patients hospitalized with CVID/XLA remained older than the non-PAD comparison group (average age, 52.0 ± 0.9 vs 49.9 ± 0.1 years; *P* < .0001) and were predominantly female (66.2%, n = 23,100). Racial distribution paralleled the broader PAD cohort, with the majority identifying as non-Hispanic White (88.5%, n = 30,060).

**Table II:** Demographic and hospitalization outcomes among CVID/XLA and non-PAD admissions, excluding obstetric admissions and standardized by age and sex (2017-20)

Characteristic	CVID/XLA admissions	Non-PAD admissions	*P* value
No., unweighted (weighted)	6,981 (34,905)	27,783,886 (1,389,194,044)	
Age (years), average [standard error]	52.0 [0.9]	49.9 [0.1]	<.0001
Sex			<.0001
Male	33.8 (11,805)	44.1 (61,271,040)	
Female	66.2 (23,100)	55.9 (77,627,759)	
Race			<.0001
Non-Hispanic White	88.5 (30,060)	64.5 (86,396,758)	
Black/African American	3.3 (1,125)	15.4 (20,666,719)	
Hispanic	5.4 (1,840)	12.7 (17,006,207)	
Other	2.7 (925)	7.3 (9,881,686)	
Total hospital cost (US$), median (IQR)	10,596 (6,368-19,646)	7,520 (3,924-14,580)	<.0001
LOS (days), median (IQR)	3.4 (1.8-6.4)	2.4 (1.3-4.7)	<.0001
Discharge disposition			<.0001
Routine	66.4 (23,160)	67.7 (94,020,501)	
Short-term hospital	1.6 (545)	2.0 (2,751,593)	
Transfer	10.8 (3,760)	13.7 (19,056,492)	
Home health care	18.6 (6,490)	13.0 (18,003,249)	
Against medical advice	0.8 (295)	1.4 (2,012,930)	
Died	1.9 (650)	2.2 (2,990,180)	.1086
Vital status			
Alive	98.1 (34,250)	97.8 (135,869,309)	<.0001
Deceased	1.9 (650)	2.2 (2,990,180)	

Data are presented as % (nos.) unless otherwise indicated.

Health care utilization patterns were consistent with the findings from the main analysis. CVID/XLA admissions had substantially higher median total hospital costs compared with non-PAD hospitalizations ($10,596 [IQR, $6,368 to $19,646] vs $7,520 [IQR, $3,924 to $14,580]; *P* < .0001) and demonstrated longer median LOS (3.4 days [IQR, 1.8-6.4] vs 2.4 days [IQR, 1.3-4.7]; *P* < .0001). Discharge patterns similarly reflected greater post–acute care needs, with home health care utilization higher in CVID/XLA admissions (18.6%, n = 6,490) compared with non-PAD hospitalizations (13%, n = 18,003,249). In-hospital mortality for CVID/XLA was 1.9% (n = 650), which was lower than mortality among non-PAD hospitalizations (2.2%, n = 2,990,180; *P* < .0001).

We further stratified PAD hospitalizations into CVID/XLA, SIGAD, and other antibody deficiencies to assess heterogeneity within the PAD cohort (see Table E3 in the Online Repository available at www.jaci-global.org). Demographic characteristics and hospitalization outcomes varied significantly across subtypes, with SIGAD representing a younger subgroup and other antibody deficiencies demonstrating the highest hospitalization costs and longest LOS.

### Hospitalization diagnoses and outcomes stratified by principal diagnosis

To characterize the clinical reasons for hospitalization among PAD patients, we examined the distribution of principal diagnosis categories across PAD subtypes (Table III). Infections accounted for a substantial proportion of admissions, ranging from 29.5% of hospitalizations among SIGAD to 43.5% among XLA (*P* < .0001). Respiratory infections represented the largest infectious subgroup, comprising 15.9% of XLA, 10.2% of CVID, 9.3% of SIGAD, and 11.6% of other PAD admissions. Sepsis and bacteremia were also common, particularly in XLA (19.1%) and CVID (14.9%). Autoimmune and autoinflammatory conditions accounted for 2.5% of XLA admissions, 1.7% of SIGAD admissions, and 1.1% of CVID admissions (Table III).

**Table III:** Distribution of primary hospitalization diagnoses among PAD subtypes (2017-20)

Diagnosis category	XLA	CVID	SIGAD	Other PAD	*P* value
Infectious diseases (overall)	43.5 (615)	30.7 (10,275)	29.5 (3,140)	34.1 (44,780)	<.0001
Respiratory infections	15.9 (225)	10.2 (3,420)	9.3 (990)	11.6 (15,305)	<.0001
Gastrointestinal infections	2.8 (40)	2.1 (700)	2.5 (265)	1.7 (2,170)	.0056
Sepsis/bacteremia	19.1 (270)	14.9 (4,985)	12.1 (1,295)	17.2 (22,605)	<.0001
Autoinflammatory/autoimmune conditions	2.5 (35)	1.1 (375)	1.7 (180)	0.9 (1,130)	<.0001
Other diagnoses	54.1 (765)	68.2 (22,840)	68.9 (7,340)	65.1 (85,505)	<.0001

Data are presented as % (nos.).

We next evaluated whether hospitalization outcomes differed by the principal clinical reason for admission (Table IV). Admissions with a gastrointestinal indication had a median hospital cost of $8,636 (IQR, $5,314 to $14,868) and a median LOS of 2.8 days (IQR, 1.6-5.0). Respiratory admissions were associated with a median cost of $8,200 (IQR, $5,047 to $14,300) and a median LOS of 3.1 days (IQR, 1.6-5.6). Hematologic and immunologic admissions had a median hospital cost of $7,359 (IQR, $4,534 to $12,496) and a median LOS of 2.6 days (IQR, 1.3-4.8) (Table IV).

**Table IV:** Hospitalization outcomes across MDC among PAD admissions (2017-20)

Characteristic	Respiratory (MDC 04)	Hematology/immunology (MDC 16)	Gastrointestinal (MDC 06)	Other MDCs	*P* value
No., unweighted (weighted)	2,595,071 (12,975,352)	343,598 (1,717,990)	2,144,542 (10,722,707)	22,736,951 (113,684,735)	
Age (years), average [standard error]	60.7 [0.2]	52.2 [0.2]	59.1 [0.1]	47.7 [0.1]	<.0001
Female, % (no.)	51.9 (6,737,423)	55.5 (953,920)	54.0 (5,793,074)	56.5 (64,247,022)	<.0001
Total hospital cost (US$), median (IQR)	8,200 (5,047-14,300)	7,359 (4,534-12,496)	8,636 (5,314-14,868)	7,295 (3,628-14,636)	<.0001
LOS (days), median (IQR)	3.1 (1.6-5.6)	2.6 (1.3-4.8)	2.8 (1.6-5.0)	2.3 (1.3-4.6)	<.0001
Routine discharge, % (no.)	16.6 (2,147,900)	11.2 (192,670)	13.4 (1,437,115)	12.6 (14,262,829)	<.0001
In-hospital mortality, % (no.)	4.1 (535,170)	1.2 (20,185)	1.4 (147,965)	2.0 (2,293,745)	<.0001

*MDC,* Major Diagnostic Category.

In-hospital mortality varied across diagnosis categories, ranging from 1.2% in hematologic/immunologic admissions to 4.1% in respiratory admissions. Routine discharge rates ranged from 11.2% among hematologic/immunologic admissions to 16.6% for respiratory diagnoses (Table IV).

### Annual trends in hospital costs and LOS

**Fig 2:**
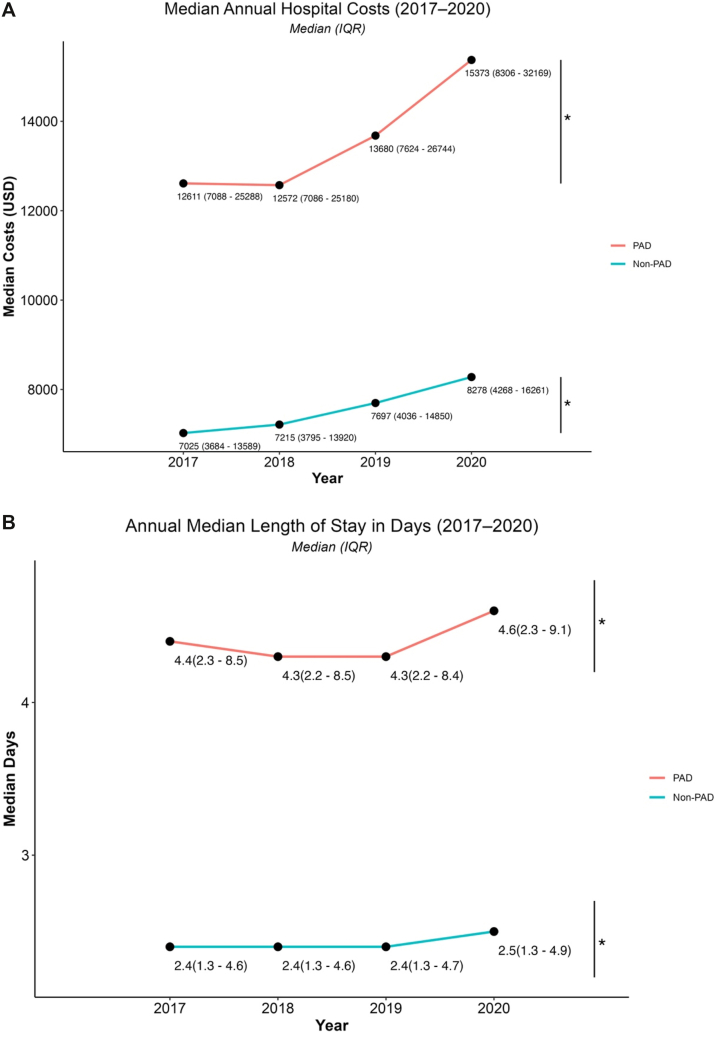
Annual trends in hospital costs and LOS for PAD vs non-PAD hospitalizations (2017-20). **A,** Median [IQR] costs for PAD admissions increased from $12,611 [$7,088 to $25,288] in 2017 to $15,373 [$8,306-32,169] in 2020. In comparison, median costs among non-PAD hospitalizations rose from $7,025 in 2017 to $8,278 in 2020. **B,** Annual median LOS. Among PAD-associated hospitalizations, median LOS remained stable from 2017 to 2019 (4.4, 4.3, and 4.3, respectively) before increasing to median [IQR] 4.6 [2.3-9.1] days in 2020. ∗*P* < .0001.

From 2017 to 2020, PAD-associated hospitalizations demonstrated a steady increase in health care resource utilization compared with non-PAD admissions (Fig 2). Median annual hospital costs for PAD hospitalizations increased from $12,611 (IQR, $7,068 to $25,288) in 2017 to $15,373 (IQR, $8,306 to $32,169) in 2020 (*P* < .0001) (Fig 2, *A*). In comparison, median costs for non-PAD hospitalizations rose from $7,025 (IQR, $3,684 to $13,589) in 2017 to $8,278 (IQR, $4,268 to $16,261) in 2020.

Parallel patterns were observed for hospital LOS (Fig 2, *B*). Median LOS among PAD hospitalizations was 4.4 days (IQR, 2.3-8.5) in 2017 and increased to 4.6 days (IQR, 2.3-9.1) in 2020 (*P* < .0001). Non-PAD admissions exhibited smaller changes over time, with median stays of 2.4 days (IQR, 1.3-4.6) in 2017 and 2.5 days (IQR, 1.3-4.9) in 2020.

### Temporal trends in discharge disposition and in-hospital mortality

**Fig 3:**
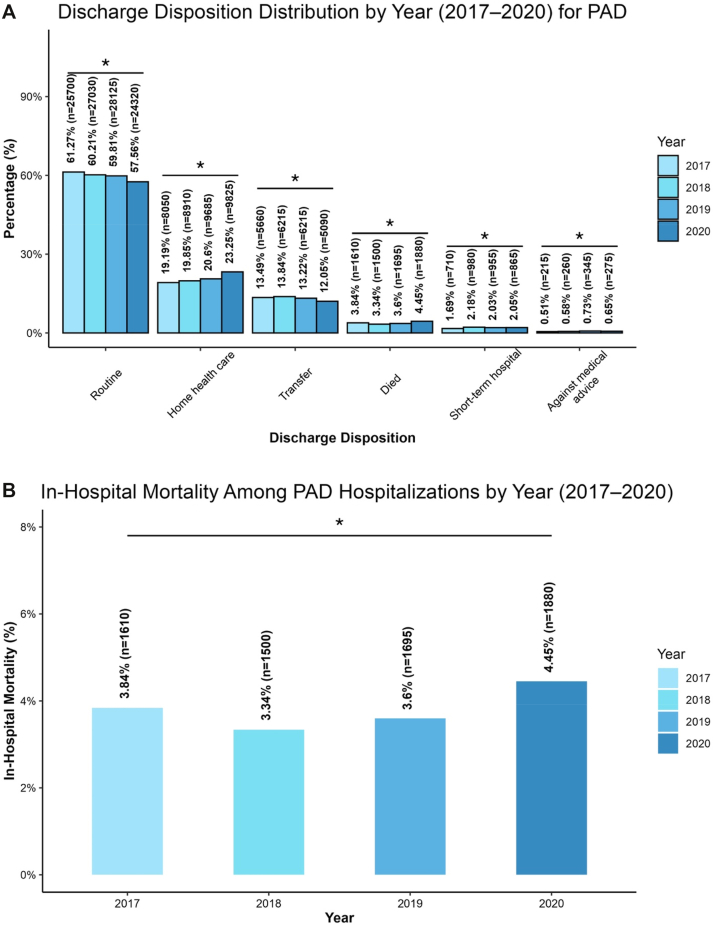
Temporal trends in discharge disposition and in-hospital mortality among PAD-associated hospitalizations (2017-20). **A,** Routine discharges decreased over time, from 61.3% (n = 25,700) in 2017 to 57.6% (n = 24,320) in 2020. Discharges to home health care increased from 19.2% (n = 8,050) in 2017 to 23.3% (n = 9,825) in 2020. ∗*P* < .0001. **B,** Annual in-hospital mortality among PAD hospitalizations, which ranged 3.8% (n = 1,610) in 2017, 3.3% (n = 1,500) in 2018, and 3.6% (n = 1,695) in 2019, rising to 4.5% (n = 1,880) in 2020. ∗*P* < .0001.

Temporal trends in discharge disposition among PAD hospitalizations from 2017 to 2020 demonstrated a progressive decline in routine home discharge (Fig 3, *A*). Routine discharges decreased from 61.3% in 2017 to 57.6% in 2020 (*P* < .0001). Over the same period, discharges to home health care services increased from 19.2% in 2017 to 23.3% in 2020. Transfers to other facilities remained relatively consistent, ranging from 13.5% to 12.1%, and discharges against medical advice remained relatively stable over the study period.

In-hospital mortality among PAD admissions showed a statistically significant upward trend across the study period (Fig 3, *B*). Mortality rates were 3.8% (n = 1,610) in 2017, 3.3% (n = 1,500) in 2018, 3.6% (n = 1,695) in 2019, and 4.5% (n = 1,880) in 2020 (*P* < .0001).

### Impact of COVID-19 on 2020 hospitalization outcomes

Given the observed worsening in costs, LOS, and in-hospital mortality in 2020, we examined whether these changes were driven by COVID-19–associated hospitalizations (Table V). In 2020, most PAD admissions did not include a COVID-19 diagnosis (n = 8,116; weighted n = 40,580), whereas 524 PAD admissions (weighted n = 2,620) were COVID-19 related.

**Table V:** Hospitalization outcomes in PAD and non-PAD patients with and without COVID-19 (2020)

Characteristic	Without COVID-19			With COVID-19		
	PAD	Non-PAD	*P* value	PAD	Non-PAD	*P* value
No., unweighted (weighted)	8,116 (40,580)	6,129,022 (30,645,112)		524 (2,620)	333,503 (1,667,515)	
Total hospital cost (US$), median (IQR)	15,171 (8,225-31,570)	8,106 (4,165-15,973)	<.0001	18,293 (9,402-38,825)	11,609 (6,611-21,908)	<.0001
LOS (days), median (IQR)	4.5 (2.3-8.9)	2.4 (1.3-4.8)	<.0001	6.2 (3.4-12.6)	4.8 (2.5-9.2)	<.0001
Routine discharge, % (no.)	23.5 (9,540)	14.0 (4,284,929)	<.0001	16.6 (435)	13.0 (216,375)	.0006
In-hospital mortality, % (no.)	3.6 (1,475)	2.2 (672,065)	<.0001	16.4 (430)	13.7 (227,830)	.0537

Outcomes were markedly worse for COVID-19–associated hospitalizations in both groups. Among PAD patients with COVID-19, median hospital cost was $18,293 (IQR, $9,402 to $38,825) and median LOS was 6.2 days (IQR, 3.4-12.6), compared with $11,609 (IQR, $6,611 to $21,908) and 4.8 days (IQR, 2.5-9.2), respectively, among non-PAD patients with COVID-19 (*P* < .0001). In-hospital mortality was substantially higher in COVID-19–associated than non-COVID-19 admissions for both groups: 16.4% (n = 430) for PAD and 13.7% (n = 227,830) for non-PAD (*P* = .0537).

Among non–COVID-19 hospitalizations, PAD continued to demonstrate higher resource utilization than non-PAD admissions. Median total hospital costs were $15,171 (IQR, $8,225 to $31,570) for PAD versus $8,106 (IQR, $4,165 to $15,973) for non-PAD (*P* < .0001), and median LOS was 4.5 days (IQR, 2.3-8.9) versus 2.4 days (IQR, 1.3-4.8), respectively (*P* < .0001). Routine discharge was more frequent among PAD than non-PAD admissions (23.5% [n = 9,540] vs 14.0% [n = 4,284,929]; *P* < .0001), yet in-hospital mortality remained higher in PAD (3.6% [n = 1,475] vs 2.2% [n = 672,065]; *P* < .0001).

### Respiratory principal diagnoses in 2020

To further explore the contribution of respiratory disease to the 2020 hospitalization burden, we evaluated admissions with a respiratory condition listed as the principal diagnosis (see Table E4 in the Online Repository available at www.jaci-global.org). Respiratory principal diagnoses were substantially more common among PAD hospitalizations than among non-PAD hospitalizations in 2020 (18.5% [n = 7,980] vs 5.9% [n = 1,891,600]; *P* < .0001). Within this respiratory stratum, PAD admissions were associated with greater health care utilization, including a longer median LOS (4.3 days; IQR, 2.4-7.5 vs 3.0 days; IQR, 1.6-5.4; *P* < .0001) and higher median total hospital costs compared with non-PAD admissions. In-hospital mortality among respiratory admissions was similar between groups, with 3.1% (n = 245) in PAD and 4.0% (n = 76,135) in non-PAD hospitalizations (*P* = .05).

### Differential impact of PAD on hospitalization outcomes in COVID-19 versus non–COVID-19 admissions

To determine whether the effect of PAD differed between COVID-19 and non–COVID-19 admissions in 2020, we evaluated adjusted associations for LOS, total hospital costs, and in-hospital mortality, including a PAD × COVID-19 interaction term (see Table E5 in the Online Repository available at www.jaci-global.org). COVID-19 significantly modified the relationship between PAD and health care utilization. Among non–COVID-19 admissions, PAD was associated with a longer adjusted LOS (GLM gamma/log coefficient 0.610; 95% CI, 0.591-0.628; *P* < .0001), whereas the corresponding effect was (coefficient 0.230; 95% CI, 0.157-0.302; *P* < .0001) in COVID-19 admissions.

A similar pattern was observed for hospitalization costs. In non–COVID-19 admissions, PAD was associated with higher adjusted total hospital costs (coefficient 1.071; 95% CI, 1.049-1.092; *P* < .0001), and (coefficient 0.490; 95% CI, 0.406-0.573; *P* < .0001) in COVID-19 admissions. In contrast, COVID-19 did not modify the association between PAD and in-hospital mortality. The adjusted effect of PAD on mortality was comparable in non–COVID-19 admissions (odds ratio, 0.77; 95% CI, 0.68-0.88; *P* < .0001) and COVID-19 admissions (odds ratio, 0.70; 95% CI, 0.56-0.88; *P* = .002), with no significant interaction (*P* = .857).

## Discussion

In this nationally representative analysis of US hospitalizations, we characterized patterns of inpatient utilization and clinical outcomes among patients with PAD using the NIS, the largest all-payer inpatient database in the United States. We also analyzed trends from 2017 to 2020, comparing PAD with non-PAD–associated hospitalizations to characterize differences in demographic profiles, hospital costs, LOS, discharge disposition, and in-hospital mortality. Despite accounting for a small proportion of total hospitalizations, PAD admissions were associated with substantially greater clinical complexity and economic burden.

PAD-associated hospitalizations demonstrated markedly increased health care utilization compared with non-PAD admissions. Median total hospital costs were substantially higher among PAD encounters ($13,961 [IQR, $7,715 to $28,438] vs $9,436 [IQR, $5,414 to $16,943]; *P* < .0001), and patients with PAD had significantly longer hospitalizations (median, 5.0 days [IQR, 2.8-9.3] vs 3.5 days [IQR, 2.0-5.8]; *P* < .0001). These national findings likely reflect the underlying clinical complexity of PAD, which is frequently complicated by recurrent or severe infections, chronic lung disease (including bronchiectasis), autoimmune and inflammatory manifestations, and other comorbidities requiring extensive diagnostic evaluation and multidisciplinary inpatient management.^8^, ^9^, ^10^, ^11^, ^12^ While earlier reports from single-center cohorts and immunology registries such as BIPAD^13^ have described hospitalization patterns in PAD, this study provides the first population-level confirmation of the substantial inpatient resource demands associated with PAD across the United States.

Hospital outcomes were consistently worse among patients with PAD. Routine discharge to home occurred in only 58.1% of PAD hospitalizations, compared with 59.6% of non-PAD admissions. PAD patients were also more frequently discharged to home health care services (21.2% vs 16.2%), reflecting greater post–acute care needs. Transfers to other facilities remained common in PAD hospitalizations (14%), though slightly less frequent than in non-PAD admissions (17.5%). In-hospital mortality was significantly higher among PAD admissions, occurring in 4.1% of hospitalizations compared with 2.7% in the non-PAD group (*P* < .0001).

Our sensitivity analyses further confirmed these findings. Excluding elective admissions had minimal impact on cohort composition or outcome patterns, as elective encounters represented a small fraction of overall hospitalizations in both groups. Regardless of whether elective admissions were included or excluded, PAD hospitalizations consistently demonstrated higher median hospital costs, longer LOS, and greater in-hospital mortality compared with non-PAD admissions. These results reinforce that the increased inpatient burden observed among PAD patients is not driven by case-mix differences in elective versus urgent admissions but instead reflects the intrinsic clinical complexity of PAD-related hospitalizations.^17^

Adjusted analyses accounting for demographic, clinical, and hospital-level characteristics provided additional insight into the independent contribution of PAD to hospitalization outcomes. After multivariable adjustment, PAD remained strongly associated with increased health care utilization, including significantly longer hospital stays and substantially higher total costs. These associations persisted even after controlling for age, sex, race/ethnicity, payer status, comorbidity burden, hospital characteristics, and admission type. In contrast, PAD status was associated with lower adjusted odds of in-hospital mortality, suggesting that the higher crude mortality observed in unadjusted comparisons may reflect confounding by comorbidity burden or other patient- or hospitalization-level factors.

Subtype-restricted analyses further supported these conclusions. Limiting the cohort to patients with CVID or XLA yielded a demographic and outcome profile consistent with the main analysis, including higher hospitalization costs, longer stays, and greater post–acute care needs relative to non-PAD hospitalizations. These validate the primary findings and highlight heterogeneity across PAD subgroups, with SIGAD and other antibody deficiencies demonstrating distinct demographic and resource utilization patterns. These findings may reflect greater illness severity at presentation, delayed recognition of immunologic disease, or challenges in inpatient management when immunology expertise is not routinely involved.^18^ They further underscore the need for strengthened discharge planning, coordinated outpatient follow-up, and early engagement of subspecialty care, particularly given that immunoglobulin replacement therapy is delivered primarily in the outpatient setting.^19^^,^^20^

Stratification by principal diagnosis provided additional insight into the drivers of hospitalization burden in PAD. Infectious diseases accounted for a substantial proportion of admissions across PAD subtypes, with particularly high contributions from respiratory infections and sepsis—patterns consistent with the hallmark susceptibility of PAD to recurrent sinopulmonary disease and systemic bacterial infections.^8^, ^9^, ^10^, ^11^, ^12^ XLA and CVID demonstrated the greatest infectious burden, aligning with prior literature describing more severe phenotypes in these disorders.^3^^,^^4^^,^^8^^,^^13^

When examining outcomes across principal diagnostic categories, respiratory admissions were associated with the longest hospital stays and highest in-hospital mortality, whereas gastrointestinal and hematologic/immunologic admissions showed comparatively lower mortality and shorter stays. These variations suggest that the clinical context of admission, particularly the presence of acute respiratory illness, substantially influences resource utilization and outcomes in PAD. Together, these findings highlight the need for heightened inpatient vigilance and targeted management strategies for PAD patients presenting with respiratory or systemic infectious illnesses, which continue to represent a major source of morbidity in this population.

Across the study period, PAD hospitalizations demonstrated a progressive increase in health care utilization and worsening clinical outcomes. Median hospitalization costs rose from $12,611 in 2017 to $15,373 in 2020, and median LOS increased from 4.4 to 4.6 days, representing a steady escalation disproportionate to the more modest changes observed in non-PAD admissions. Concurrently, routine discharge rates declined while reliance on home health services increased, reflecting growing post–acute care needs in this population. Most notably, in-hospital mortality rose from 3.8% to 4.5% between 2017 and 2020. These trends likely reflect a combination of increasing disease complexity and broader health care system pressures, but the marked inflection in 2020 aligns with the onset of the COVID-19 pandemic. Patients with inborn errors of immunity, including PAD, have been shown to experience more severe COVID-19 outcomes,^21^^,^^22^ and disruptions in routine outpatient management, such as delayed immunoglobulin infusions or reduced access to specialty care, may have further contributed to the worsening inpatient profile observed during the pandemic year.

Among hospitalizations involving COVID-19, outcomes were substantially worse in both PAD and non-PAD patients, consistent with the significant clinical burden of severe acute respiratory syndrome coronavirus 2 in immunologically vulnerable populations.^21^^,^^22^ PAD patients with COVID-19 experienced significantly higher costs and longer LOS than their non-PAD counterparts, suggesting increased illness severity when immunodeficiency coexists with viral infection. In-hospital mortality approached 16% among PAD patients—a profoundly elevated rate, even if not statistically different from the mortality observed in non-PAD COVID-19 admissions.

Respiratory conditions contributed meaningfully to the overall 2020 hospitalization burden. Respiratory principal diagnoses were more than 3 times as common among PAD patients as among non-PAD patients, and within this respiratory stratum, PAD hospitalizations were again associated with longer stays and higher costs. Interestingly, mortality in respiratory admissions did not differ significantly by PAD status, suggesting that acute respiratory illnesses themselves, may be the predominant drivers of short-term mortality in this subgroup.

Interaction analyses provided further clarity regarding the relationship between PAD and COVID-19. COVID-19 significantly modified the association between PAD and health care utilization. The excess burden of PAD on LOS and hospitalization costs was more pronounced in non–COVID-19 admissions than in those involving COVID-19. This attenuation likely reflects the uniformly high severity and prolonged course of COVID-19 hospitalizations, which may overshadow baseline utilization differences attributable to PAD alone. In contrast, COVID-19 did not modify the association between PAD and mortality. Together, these findings suggest that while PAD is consistently associated with increased health care utilization, pandemic-related shifts in patient composition and disease severity contributed substantially to the disproportionate rise in hospitalization burden observed in 2020.

Key strengths of our study include the use of the NIS, which is a large, nationally representative dataset, and comprehensive survey-weighted analyses that allowed robust comparisons between PAD and non-PAD hospitalizations. The incorporation of multiple sensitivity analyses, including exclusion of elective admissions, restriction to CVID/XLA, stratification by principal diagnosis category, and COVID-19–specific analyses, strengthens the validity of our findings and provides important context regarding heterogeneity within the PAD population. Evaluation of temporal trends from 2017 to 2020, combined with interaction modeling to assess the modifying effect of COVID-19, offers a nuanced understanding of how health care utilization and outcomes evolved during both prepandemic and pandemic years. By leveraging nationally representative data across diverse geographic and demographic settings, our study provides the most comprehensive population-level assessment to date of the inpatient burden associated with PAD.

However, there were several limitations to our study. The reliance on administrative data inherently limits clinical granularity because information regarding PAD subtype, immunoglobulin therapy receipt, infection etiology, laboratory data, and outpatient treatment is not captured. The use of ICD coding is also a limitation, as diagnosis codes may be subject to variability and misclassification. Additionally, because the NIS lacks person-level identifiers, we were unable to distinguish unique individuals or evaluate outcomes such as readmissions or longitudinal progress. The NIS lacks person-level identifiers, precluding assessment of readmissions, longitudinal disease trajectories, or cumulative hospitalization burden. COVID-19 severity measures (eg, intensive care unit admission, oxygen requirements) are not available, limiting interpretation of pandemic-related findings. Additionally, the cross-sectional design prevents causal inference. Despite these limitations, the consistency of results across multiple sensitivity and subgroup analyses increases confidence in the robustness of our conclusions and highlights important opportunities to improve inpatient and transitional care for PAD.

In conclusion, PAD-associated hospitalizations, though relatively uncommon, impose a substantial clinical and economic burden on the US health care system. PAD admissions were marked by significantly higher costs, longer hospital stays, greater reliance on post–acute care services, and elevated in-hospital mortality in unadjusted analyses. Even after extensive adjustment, PAD remained independently associated with increased health care utilization. Temporal trends demonstrated progressive worsening from 2017 to 2020, with the greatest inflection during the COVID-19 pandemic. Analyses stratified by COVID-19 status revealed markedly poorer outcomes in COVID-19–associated admissions for both PAD and non-PAD patients, and interaction models demonstrated that COVID-19 modified the relationship between PAD and hospitalization resource use.

Strengthening early recognition, outpatient management, immunology collaboration, and transitional care pathways may reduce preventable hospitalizations and improve outcomes for this medically complex population. Future research is needed to link administrative data with immunology registries such as the United States Immunodeficiency Network or electronic health records to capture detailed clinical variables including laboratory data, treatment trajectories, and longitudinal outcomes. Interventional studies evaluating the effectiveness of integrated care models, including early immunology consultation, structured case management, and follow-up after discharge, are needed to reduce inpatient burden.

**Clinical implication**

These findings highlight the importance of proactive PAD management strategies, including timely diagnosis and structured care coordination, to optimize patient outcomes.

## Disclosure statement

S.B. is supported by the 10.13039/100000060National Institute of Allergy and Infectious Diseases of the 10.13039/100000002National Institutes of Health (NIH; award K23AI163350) and a faculty development award from the American Academy of Allergy, Asthma & Immunology. The content is solely the responsibility of the authors and does not necessarily represent the official views of the NIH.

Disclosure of potential conflict of interest: S. Barmettler has served as a consultant for CSL Behring, Octapharma, Takeda, and Vertex; and has received investigator-initiated research grants from Bristol-Myers Squibb and Pharming. The rest of the authors declare that they have no relevant conflicts of interest.

## Supplementary data

Supplementary Fig E1
