# Supplementary figures and images for: Nationwide analysis of temporal trends and outcomes in hospitalized patients with predominantly antibody deficiency using the National Inpatient Sample
Source: J Allergy Clin Immunol Glob. 2026 Mar 20;5(3):100691. doi: 10.1016/j.jacig.2026.100691 (PMC13091990; doi:10.1016/j.jacig.2026.100691)

**FIGURES**

**Figure S1.**

**
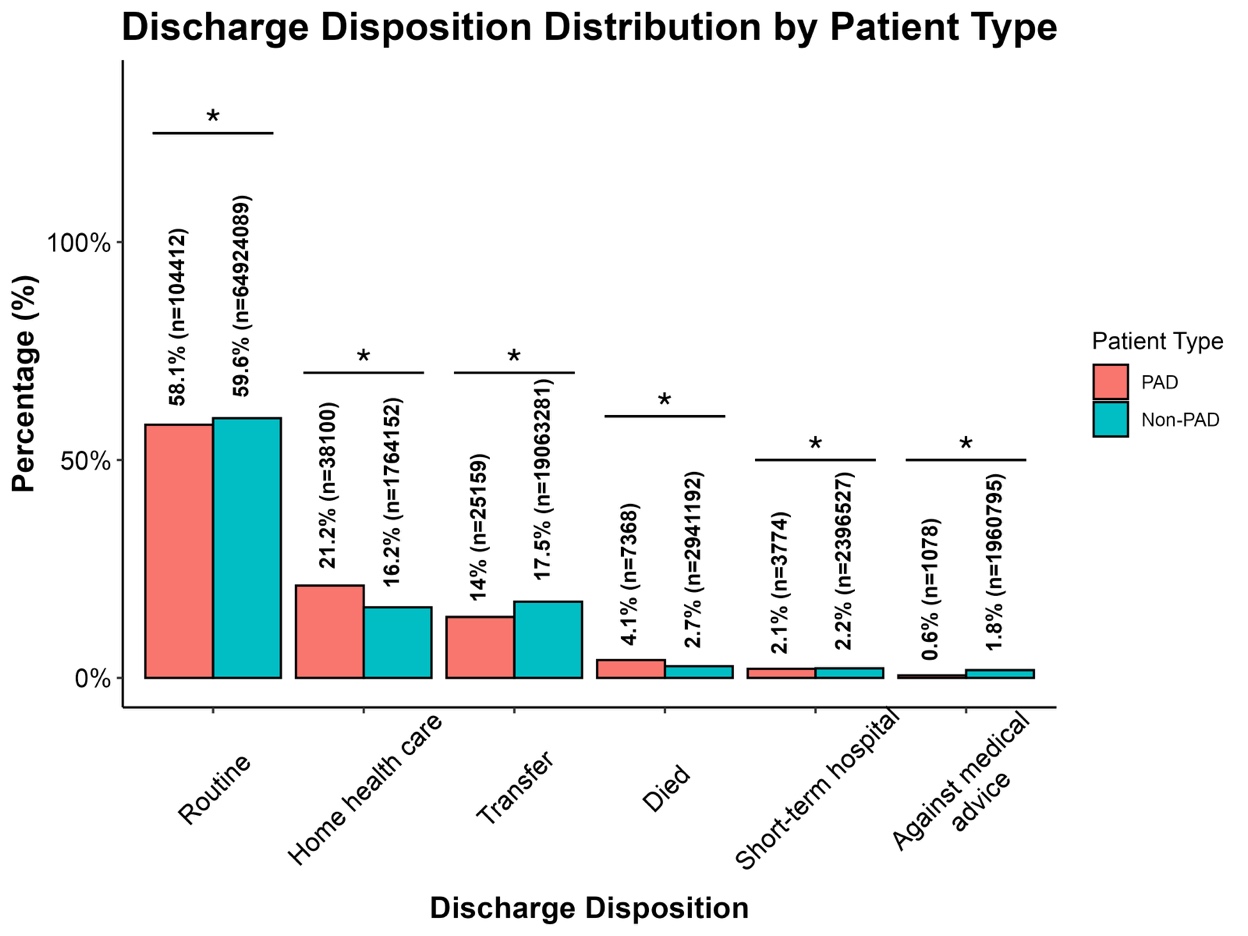
**


Supplement: Supplementary Fig E1 [file mmc1.docx]
