# Supplementary material for: Nationwide analysis of temporal trends and outcomes in hospitalized patients with predominantly antibody deficiency using the National Inpatient Sample
Source: J Allergy Clin Immunol Glob. 2026 Mar 20;5(3):100691. doi: 10.1016/j.jacig.2026.100691 (PMC13091990; doi:10.1016/j.jacig.2026.100691)

**SUPPLEMENT**

**Figure S1. Discharge Disposition and In-Hospital Mortality in PAD compared to Non-PAD Hospitalizations.**

**Figure S1 shows the distribution of discharge disposition for PAD versus non-PAD hospitalizations. PAD-associated admissions were less likely to result in routine discharge (58.1% vs. 59.6%) and more frequently required home health care (21.2% vs. 16.2%). ∗p<0.0001.**

**TABLES**

**Table S1.**

| **A) Number of elective admissions excluded from PAD and non-PAD cohorts (2017–2020).** | | | | | | |
| --- | --- | --- | --- | --- | --- | --- |
| **Group** | **Total admissions before exclusion N, unweighted (weighted)** | | **Elective admissions excluded (% [n])** | | **Final cohort size N, unweighted (weighted)** | |
| **PAD** | 36,276 (181,380) | | 14.2 (5,157) | | 31,119 (155,595) | |
| **Non-PAD** | 27,783,886 (1,389,194,044) | | 19.9 (5,514,349) | | 22,269,537 (1,113,476,666) | |
| **P-value** |  | | <0.0001 | |  | |
| **B) Sensitivity analysis of hospitalization outcomes including vs excluding elective admissions (2017–2020).** | | | | | | |
| **Outcome** | **PAD**  **(including elective)** | **Non-PAD**  **(including elective)** | **P-value** | **PAD**  **(excluding elective)** | **Non-PAD**  **(excluding elective)** | **P-value** |
| **N, unweighted (weighted)** | 36,276  (181,380) | 27,783,886 (1,389,194,040) |  | 31,119  (155,595) | 22,269,537  (1,113,476,666) |  |
| **Total hospital cost, median [IQR], $** | 13,502  (7,479–27,154) | 7,520  (3,924–14,580) | <0.0001 | 12,973  (7,310–25,934) | 6,984  (3,695–13,318) | <0.0001 |
| **Length of stay, median [IQR], days** | 4.4 (2.2–8.6) | 2.4 (1.3–4.7) | <0.0001 | 4.5 (2.3–8.6) | 2.6 (1.4–5.0) | <0.0001 |
| **In-hospital mortality, (% [n])** | 3.8 (6,885) | 2.2 (2,990,180) | <0.0001 | 4.1 (6,310) | 0.25 (2,739,225) | <0.0001 |
| **Routine Discharge, (% [n])** | 20.5 (37,265) | 1.3 (18,003,249) | <0.0001 | 20.7 (32,220) | 1.22 (13,573,554) | <0.0001 |

**Table S2. Adjusted associations between PAD status and hospitalization outcomes (2017–2020).**

| **Outcome** | **Adjusted Estimate (PAD vs Non-PAD)** | **95% CI** | **P-value** |
| --- | --- | --- | --- |
| In-hospital mortality (survey-weighted logistic regression, OR) | 0.738 | 0.694–0.785 | <0.0001 |
| Length of stay (GLM Gamma/log coefficient) | 0.537 | 0.528–0.546 | <0.0001 |
| Total hospital cost (GLM Gamma/log coefficient) | 0.938 | 0.928–0.948 | <0.0001 |
| Models adjusted for age, sex, race/ethnicity, payer, hospital bed size, hospital location, teaching status,  census region, elective admission status, and Elixhauser comorbidity indices (mortality and readmission). | | | |

**Table S3. Hospitalization outcomes among XLA/CVID, SIGAD, and other predominantly antibody deficiencies (2017–2020).**

| **Outcome** | **CVID/XLA** | **SIGAD** | **Other PAD** | **P-value** |
| --- | --- | --- | --- | --- |
| **Average age (years, [SE])** | 52.0 (0.9) | 46.2 (0.6) | 56.7 (0.4) | <0.0001 |
| **Sex (% [n])** |  |  |  | <0.0001 |
| Male | 33.8 (11,805) | 39.7 (4,235) | 45.8 (60,195) |  |
| Female | 66.2 (23,100) | 60.3 (6,425) | 54.2 (71,210) |  |
| **Race (% [n])** |  |  |  | <0.0001 |
| Non-Hispanic White | 88.5 (30,060) | 83.7 (8,625) | 82.4 (105,145) |  |
| Black/African American | 3.3 (1,125) | 6.8 (700) | 5.8 (7,410) |  |
| Hispanic | 5.4 (1,840) | 5.3 (550) | 7.1 (9,010) |  |
| Others | 2.7 (925) | 4.2 (435) | 4.7 (6,060) |  |
| **Total hospital cost, median [IQR], $** | 10,596 (6,368–19,646) | 10,691 (5,777–20,475) | 14,714 (8,125–30,306) | <0.0001 |
| **Length of stay, median [IQR], days** | 3.4 (1.8–6.4) | 3.3 (1.7–6.6) | 4.8 (2.5–9.6) | <0.0001 |
| **Discharge Disposition (% [n])** |  |  |  | <0.0001 |
| Routine | 66.4 (23,160) | 70.1 (7,470) | 57.2 (75,105) |  |
| Short-term hospital | 1.6 (545) | 1.8 (195) | 2.2 (2,850) |  |
| Transfer | 10.8 (3,760) | 11.3 (1,200) | 14.0 (18,425) |  |
| Home health care | 18.6 (6,490) | 14.4 (1,530) | 21.8 (28,700) |  |
| Against medical advice | 0.8 (295) | 0.8 (85) | 0.6 (730) |  |
| Died | 1.9 (650) | 1.7 (180) | 4.2 (5,575) |  |
| **Vital Status (% [n])** |  |  |  | <0.0001 |
| Alive | 98.1 (34,250) | 98.3 (10,480) | 95.8 (125,820) |  |
| Deceased | 1.9 (650) | 1.7 (180) | 4.2 (5,575) |  |

**Table S4. Prevalence and outcomes for respiratory principal diagnoses in 2020 Among PAD and non-PAD admissions.**

| **Metric** | **PAD** | **Non-PAD** | **P-value** |
| --- | --- | --- | --- |
| **Respiratory principal diagnosis, (% [n])** | 18.5 (7,980) | 5.9 (1,891,600) | <0.0001 |
| **N within respiratory-principal stratum, unweighted (weighted)** | 2,656 (3,0272) | 141.9 (14,013) |  |
| **Length of stay, median [IQR]** | 4.3 (2.4–7.5) | 3.0 (1.6–5.4) | <0.0001 |
| **Total hospital cost, median [IQR], $** | 4.3 (2.4–7.5) | 3.0 (1.6–5.4) | <0.0001 |
| **In-hospital mortality, (% [n])** | 3.1 (245) | 4.0 (76,135) | 0.05 |

**Table S5. Interaction between PAD status and COVID-19 in adjusted models of 2020 hospitalization outcomes.**

| **Outcome** | **Effect of PAD in non-COVID-19 hospitalizations (estimate, 95% CI)** | **P-value** | **Effect of PAD in COVID-19 hospitalizations (estimate, 95% CI)** | **P-value** | **P-value for PAD×COVID-19 interaction** |
| --- | --- | --- | --- | --- | --- |
| In-hospital mortality (survey-weighted logistic regression, OR) | 0.774 (0.682–0.878) | <0.0001 | 0.703 (0.560–0.882) | 0.002 | 0.857 |
| Length of stay (GLM Gamma/log coefficient) | 0.610 (0.591–0.628) | <0.0001 | 0.230 (0.157–0.302) | <0.0001 | <0.0001 |
| Total hospital cost (GLM Gamma/log coefficient) | 1.071 (1.049–1.092) | <0.0001 | 0.490 (0.406–0.573) | <0.0001 | <0.0001 |


Supplement: Supplementary Tables [file mmc2.docx]
